# Research on vector-borne diseases: implementation of research communication strategies

**DOI:** 10.1186/s40249-019-0610-0

**Published:** 2019-12-05

**Authors:** Thomas Scalway, Mariam Otmani del Barrio, Bernadette Ramirez

**Affiliations:** 1 Lushomo Communications, Cape Town, South Africa; 2 Unit on Vectors, Environment and Society, The UNICEF/UNDP/World Bank/WHO Special Programme for Research and Training in Tropical Diseases, World Health Organization, Geneva, Switzerland

**Keywords:** Research uptake, Communication, Evidence into action, Knowledge translation

## Abstract

**Background:**

Effective communication of research findings on vector-borne diseases in Africa is challenging for a number of reasons. Following the experiences of a number of researchers over the life of a project, this article looks for lessons that can be shared with the wider research community.

**Main body:**

Between 2014 and 2017, a set of five inter-disciplinary teams from seven African countries collaborated on a project focusing on vector-borne diseases in the context of climate change. A central objective of this work was to influence policy and programming with relevant research findings. This article examines how principles of research communication, derived from the literature and current guidelines, can be applied in practice. Several challenges and lessons are highlighted, showing that research communication takes place within difficult constraints and in complex, fluid institutional and political environments. The processes of communication between policymakers and researchers including stakeholder mapping, defining research communication plans and tailoring communication products are discussed.

**Conclusions:**

The article concludes that while guidelines and frameworks for research communication are helpful, they should not detract from the ability of local teams to adapt to circumstances. Of key importance are the relationships and networks of local research teams.

## Multilingual abstracts

Please see Additional file 1 for translations of the abstract into the five official working languages of the United Nations.

## Background

Diseases transmitted to humans by vectors account for 17% of all infectious diseases and are a significant public health concern [1]. Large scale and coordinated vector control programmes have contributed to the decline of the global mortality attributed to vector-borne diseases (VBDs). However, with environmental changes, including climate change, the impact on VBDs is anticipated to be even more significant in terms of VBD-related hazards, vulnerabilities and exposure. While there is growing awareness on the vulnerability of the African continent to VBDs in the face of climate change, and the need for evidence-informed policy is understood, scholars still struggle to replicate theoretical guidelines to evidence uptake in real world settings [2].

Research on population health vulnerabilities to VBDs and how communities in the African drylands can be more resilient to climate change is a priority theme supported by the World Health Organization (WHO) and the Special Programme for Research and Training in Tropical Diseases (TDR) and Institutional Development Research Centre (IDRC) research initiative on VBDs and Climate Change. Research communication was a key objective of each of the projects. The five projects that comprised this research initiative were conducted by researchers from institutions in Botswana, Cote d’Ivoire, Kenya, Mauritania, South Africa, Tanzania and Zimbabwe, and included four VBDs: malaria, schistosomiasis, human African trypanosomiasis and Rift Valley fever. Investigators took a transdisciplinary, socioecological systems (SES) approach to reveal how environmental and socio-economic changes affect transmission dynamics and disease burden of VBDs through changes in vector ecology, human ecology, social organization, demography and health systems. Through this research initiative, the projects aimed to improve the capacity of African researchers and institutions to generate, analyse and use climate, environmental and socio-economic information to guide adaptive disease prevention and control strategies. The goal was to share better approaches to VBD risk management and health adaptation to climate change, especially for vulnerable populations, with their respective countries’ policymakers.

One of the important aspects of this research initiative was to embed targeted communication with stakeholders in all phases of the research projects’ implementation – from research design through to research uptake. This stems from the principle that interaction between researchers and decision-makers should happen from the inception of research projects, and should continue throughout the project duration. One of the assumptions underlying the research initiative was that evidence produced during the course of the research project needs to be communicated to stakeholders on a regular, iterative basis in order to facilitate and enable research uptake. These research communication principles were embedded in the original project design and the experience of their implementation is discussed here.

The information in this paper is condensed from continued collaboration and discussions with researchers from the five projects over the duration of the study. Interviews were undertaken with the principal investigators of the projects. These were supplemented by interviews with four policymakers from the ministries of health and ministries of environment from the respective countries, and who attended a research uptake meeting in Brazzaville in April 2017, organised by TDR/IDRC. Interviews were face-to-face and telephonic, and followed a semi-structured format. Recordings and notes were analysed and key themes were identified, which are documented here.

### Research communication strategies

Research uptake activities include making useful and relevant research available to decision-makers and ensuring that they are willing and have the capacity to use it. In recent years, there has been a growth in interest in evidence-informed policymaking, as shown by the proliferation of agencies, events, projects and journal articles focusing on this area [2] and numerous approaches have been developed to understand and facilitate the use of research evidence in policy [3]. Despite this, as stated by Georgalakis et al. [4], “Put simply, the development sector has continued to struggle to repeat the trick of turning research into action.” One of the reasons for this is that knowledge translation strategies do not always account for the specific context or the complexity of research or policymaking, especially in low- and middle-income countries [5].

There are numerous ways of understanding the objectives of research communication and what one can hope to achieve. The United Kingdom’s international development agency, the Department for International Development (DFID), outlines a straightforward approach to communicating research through their document “Research Guidance: A guide for DFID Programmes”. In this piece, research communication is defined as “*the process of interpreting or translating complex research findings into a language, format and context that non-experts can understand. It goes way beyond the mere dissemination of research results. It involves a network of participants and beneficiaries. Researchers themselves, journalists, editors and their media, intermediaries who provide links between stakeholders: all these form an interdependent network linking their differing roles in the communication process*” [6].

This paper considers the experience of applying some of the recommendations from the research uptake literature throughout the course of this TDR/IDRC research initiative. While the communities that the research teams worked with were arguably the most important stakeholders for communication, for brevity and focus, this article focuses mainly on the interface between research and policy/programming.

The frameworks and models used for research uptake include a number of common principles: undertaking stakeholder mapping to establish who is important to reach with research communication; developing a research uptake plan linked to specific communication objectives; developing a range of communication products linked to communication objectives, and the importance of early, sustained and responsible communication between researchers and decision-makers.

In the following discussion we will look briefly at the experience of endeavouring to adopt each of these principles throughout the TDR/IDRC research initiative. What is revealed in this process is that broader issues relating to divides in institutional culture, the way that research programmes originate and are funded, and a number of situational factors relating to different research sites and settings, can all have a profound impact in putting research communication principles into practice.

#### Undertaking stakeholder mapping to establish who is important to reach with research communication

Efforts to influence policy and programming through research can be improved by generating a clear picture of who are the key actors to influence. According to Georgalakis et al., “It would require social network analysis in most cases to really understand research-to-policy processes and how things actually get done” [4]. This includes looking at the people involved in research and policy, the connections between them, individual and group interests and proclivities. Numerous studies and frameworks, such as the RAPID CEL Framework, note the importance of looking at how people and networks affect the evidence into policy process [7]. Despite the importance of strong communication channels and links between researchers, policymakers and implementers, these channels are weak in many cases.

Georgalakis (2015) [8] stressed the importance of understanding policy, power relations and knowledge contexts. This involves understanding the environment within which we want change to occur, and mapping out desired changes, key stakeholders and policy processes. This can facilitate “engaging with the politics of knowledge”, rather than merely producing and disseminating knowledge [8].

To try to influence policy, whole systems should be tackled [9], and stakeholder mapping should therefore extend to cover a landscape of politically-affiliated actors who together can make a difference in policy and programming. In the case of this multi-disciplinary project, government stakeholders were from multiple sectors, including environment, health, veterinary departments and national meteorological services. Stakeholder groups included in research uptake plans by the research teams included the public sector, civil society, local community groups, private sector and international organisations. Key actors within these groups included parliamentarians, civil servants, local government representatives and officials, local councils, media, faith groups, advocacy groups, non-governmental organizations, business leaders and corporations. Although researchers were aware of the relevance of their work to many communities, there was difficulty in determining who to prioritise in their research uptake plans. The projects demonstrated the need to look beyond producing evidence to also include looking at the demand for evidence from policymakers.

While stakeholder mapping may not be as systematic or comprehensive for the TDR/IDRC research initiative as desired, the institutional contact points needed to move the projects forward were well-considered. The research teams were adept at getting their projects approved and supported by government. The researchers actively identified and communicated with people in diverse government departments at national and local levels, and engaged in a number of regional and international fora. Stakeholder mapping approaches did not follow a uniform structure or externally imposed principles and guidelines, but their localization and adaptive style may also have been a strength in terms of ensuring the projects could find engagement with the right people in the right places.

#### Developing a research uptake plan linked to specific communication objectives

The tools and recommendations for research uptake generally include a process where a plan is constructed that is tied to specific communication objectives. This research uptake plan would address an area of policy or programming where a change can be made to improve health outcomes in the area of VBDs and climate change. This involves a process in which research questions are tailored, preferably together with policymakers, and then research approaches and research uptake plans would follow on from those questions.

Developing a research uptake plan entails looking at the interaction of knowledge, policy, power relations and gender dynamics [10]. For greatest effectiveness, research uptake plans would be integrated with the project activities early in the initiative’s inception.

All the research teams in our programme had some elements of this idealised approach, but in each case it was shaped by a range of situational factors, histories and networks. The teams differed widely in how problems were defined, issues constructed, and approaches framed according to local factors.

All the teams noted in their proposals that it was important that the results of the work be used to create awareness amongst communities and national governments and that they would aim to have some impact on programming or policy. Beyond this, the specific outcomes that the projects were aiming for were not defined. Outcome-related language was used by researchers interviewed and in technical reports, but not in relation to specific communication activities. One reason that was cited by many of the researchers was that they themselves were not communication experts, and they had to prioritize their research activities over intensive research communication planning. They welcomed efforts to support their communications, and it may be useful to give further consideration to the types of support required by researchers as they embark on projects where policy influence and evidence uptake is a desired outcome.

#### Developing a range of communication products linked to communication objectives

The principle that communication products should be linked to communication objectives rooted in a coherent research uptake strategy is found in many tools, with “poor communication and dissemination” having been identified as a barrier to evidence uptake [11]. In line with this, communication and dissemination strategies including policy briefs, dialogues, and knowledge translation platforms are used as ways to improve research uptake. However, the evidence of the effectiveness of the different knowledge transfer strategies is limited [12] and how to optimally engage decision-makers is an ongoing matter for debate [13]. This is especially the case in low- and middle-income countries, where there is little research on engagement strategies that support the synthesis of policy-relevant knowledge, making the selection of optimal knowledge transfer strategies difficult [13].

The five TDR/IDRC research projects shared the results and insights of their work through a diverse range of channels: publishing in scientific journals, presenting at local scientific conferences, disseminating policy and action briefs, engaging with local media, and holding stakeholder meetings. Research findings and progress updates were shared through a website (vbd-environment.org) and also in a final research uptake meeting that brought policymakers and researchers together. At a national level, communication outputs included a range of information, education and communication (IEC) materials, radio shows, movies, local theatre, high-level national policy fora, road-shows, working with print, broadcast media, social media, online publicity and more. While this eclectic mix of channels may show that the research teams were adept at communicating to a range of different audiences, it also raises questions around how different communication channels were prioritised when there was no research uptake plan elaborated.

The TDR/IDRC researchers noted that the technical and focused format of their findings was inappropriate for non-academic audiences and that engaging with decision-makers to discuss findings was beneficial. Policymakers who attended the TDR/IDRC workshop noted that they found information sharing sessions useful and that these were most beneficial when there was plenty of time to interact with the researchers and gain an understanding of their work. These workshops were one way that the project supported the capacity of policymakers to engage with research, which a variety of commentators note as important [5, 14].

Researchers mentioned that the priorities of the government were often very different from their own. Government representatives were frequently dealing with emergencies, including outbreaks of disease such as Ebola, or in terms of broader social and economic challenges, for example the collapse of the economy in Zimbabwe. Policymakers were often only in post for short periods of time, and policy and programming agendas were often shifting in the different national settings. In this context, it was sometimes difficult to attract and maintain the attention of policymakers on the research undertaken. Other commentators have noted how national agendas might not be receptive of scientific findings from researchers. For example, according to Tyler [15], economics and law are usually given preference over scientific evidence, and public opinion may matter as much as the quality of research in shaping policy. In Oliver et al.’s (2014) [14] review of barriers to and facilitators of research uptake, competing pressures (economic, political, social, and cultural) were seen as important factors influencing the use of evidence in decision-making.

As a last point on communication products, one of the demands made on many researchers is to publish in peer-reviewed academic journals–to “publish or perish”. Many of the researchers were therefore concerned with sharing their findings through journal articles. Researchers were reluctant to share any specific findings through other channels before their research had been published as they said this would jeopardise their ability to be published. Over the course of the projects, it became clear that key findings could be released in summary form for non-academic audiences without endangering the publication process. Such a workaround shows the kinds of practical guidance that might be useful to those embarking on these and similar projects.

#### The importance of early, sustained and responsible communication between researchers and policymakers

Research suggests that ongoing iterative collaboration between researchers and policymakers can facilitate the evidence-to-policy process. “Rather than viewing users as passive recipients of information, effective research communication includes them in shaping the research and encourages their input throughout the research cycle” [6]. As stated previously, networks and partnerships are regarded by many as being a primary factor in influencing research uptake, with a number of studies focusing on the co-production of knowledge between researchers and decision-makers.

Within the TDR/IDRC research initiative there was substantial communication between the researchers and government stakeholders, which sometimes took place within long term relationships that far preceded the project. In a number of instances researchers were already conducting work in communities and working closely with local government partners.

In some cases, researchers were part of government facilities, while policymakers had become an active part of the academic community in other cases (for example studying for their own PhD). In these instances, research questions were more likely to be jointly asked and answered in a way that is valuable to both the researchers and decision makers. Other commentators have noted that when policymakers are involved in designing and implementing research, evidence uptake is more likely to occur [5]. These examples emphasize how researchers are social actors, communicating their knowledge in different formal and informal ways and key areas in the importance of knowledge transfer relate to networks and partnerships and modes of brokerage [4].

While this early communication and web of pre-existing relationships existed, they were not incorporated as part of the narrative around research uptake and were almost accidental to the programme design. The research projects also relied on government infrastructure for assistance. Government research facilities were used by some of the teams, for example the Rekomitjie Research Centre was used for the study of tsetse flies in Zimbabwe. In Cote d’Ivoire, government supplies were provided by the national schistosomiasis programme for the treatment and management of schistosomiasis cases that were identified through the household testing component of the research project. National and local government officials also contributed in the design, development and testing of household surveys that were used by the research teams. In these examples and others, there is evidence of strong collaboration and intensive communication between researchers and policymakers. Echoing those looking at the importance of engaging with the specific social and political context, the project under discussion suggested that it was these ongoing, complex relationships between researchers and those shaping policy and programming that seemed to have the most impact in terms of turning evidence into action [4]. The central importance of relationships, networks and trust between researchers and policymakers does suggest that it is this quality of relationship and local interaction that should take priority over pre-packaged formulas for research communication.

## Conclusions

The experience of this project highlights the value of many research communication theories and frameworks, but also suggests that approaches should not be too formulaic or externally imposed. Although, for the most part, stakeholder engagement strategies and communication plans were not clearly detailed in the research plans, the researchers already had advanced insights into how to work with those in the policy domain in order to get their research approved and supported.

To date, the project teams are still informally tracking the policy influence of this project 2 years after the activities end and it would be useful to intensify this monitoring further. For research on VBDs and other diseases of poverty to impact policymakers it is useful to ensure there are practical communication measures in place to address the gaps between researchers and decision-makers. These measures need to commence at project inception, and should include building on the pre-existing local networks and relationships of senior researchers, supporting direct engagement between researchers and decision-makers. An important recommendation arising from the experiences documented above is that funding earmarked for research communication is important to enable researchers to share their findings with those able to act upon them.

## Supplementary information

**Additional file 1.** Multilingual abstracts in the five official working languages of the United Nations.

## Data Availability

Data sharing is not applicable to this article as no datasets were generated or analysed during the current study.

## Abbreviations

DFID: Department for International Development
IDRC: International Development Research Centre
SES: Socioecological systems
TDR: Special Programme for Research and Training in Tropical Diseases
VBD: Vector-borne disease
