# Supplementary material for: Research on vector-borne diseases: implementation of research communication strategies
Source: Infect Dis Poverty. 2019 Dec 5;8:101. doi: 10.1186/s40249-019-0610-0 (PMC6896314; doi:10.1186/s40249-019-0610-0)
Supplement: Supplementary file 1 — Additional file 1. Multilingual abstracts in the five official working languages of the United Nations. [file 40249_2019_610_MOESM1_ESM.pdf]

Translation of the abstract into the five official working languages of the United Nations

بحث حول الأمراض المنقولة بالحشرات: تنفيذ استراتيجيات التواصل البحثي

توماس سكالواي، ومريم عثماني ، دل باريو، وبرناديت راميريز

نبذة مختصرة
معلومات عامة : يمثل الإبلاغ الفعال عن نتائج الأبحاث المتعلقة بالأمراض المنقولة بواسطة الحشرات في إفريقيا تحديًا لعدة أسباب. و بناء على خبرات عدد من الباحثين على طول امتداد فترة مشروع ما ، تبحث هذه المقالة عن دروس يمكن مشاركتها مع الوسط البحثي الأوسع.
المتن الرئيسي: بين عامي 2014 و 2017 ، تعاونت مجموعة من خمسة فرق متعددة التخصصات من سبع دول إفريقية على مشروع يركز على الأمراض المنقولة بواسطة الحشرات في سياق تغير المناخ. كان الهدف الرئيسي لهذا العمل هو التأثير على السياسات و وضع برامج ذات صلة بالنتائج البحثية. و تتناول هذه المقالة كيفية تطبيق مبادئ التواصل البحثي ، والمستمدة من الأدبيات والمبادئ التوجيهية الحالية في الممارسة العملية. و تم تسليط الضوء على العديد من التحديات والدروس ، التي تبين أن التواصل البحثي يتم في ظل قيود صعبة وفي بيئات مؤسساتية وسياسية معقدة. كما تمت مناقشة عمليات الاتصال بين واضعي السياسات والباحثين، بما في ذلك تعيين أصحاب المصلحة ،تحديد خطط التواصل البحثي و تعديل منتجات الاتصالات.
الإستنتاجات : تخلص المقالة إلى أنه على رغم كون الإرشادات والأطر الخاصة بالاتصال البحثي مفيدة ، يجب ألا تنتقص من قدرة الفرق المحلية على التكيف مع الظروف. و أن العلاقات و الشبكات الخاصة بالفرق البحثية المحلية هي ذات أهمية قصوى.

Translated from English version into Arabic by Hamid Alahmari, revised by Mohamed Aberzak, through

TRANSLATORS WITHOUT BORDERS

媒介传播疾病的研究：研究交流策略的实施

Thomas Scalway, Mariam Otmani del Barrio and Bernadette Ramirez

摘要
引言：许多原因导致有效传播非洲的媒传疾病的研究结果成为一个挑战。根据许多研究人员在项目实施过程中取得的经验，本文拟寻找可与更广泛的研究群体共享的经验与教训。
主要内容：2014–2017 年，来自七个非洲国家的五个跨学科小组合作开展了一个项目，重点关注气候变化背景下的媒传疾病。该项目的主要目标是通过相关的研究结果来影响政策和计划制定。本文研究了如何从文献和当前指南中得出研究交流的原理，并将其应用于实践。强调了一些挑战和教训，表明研究交流是在困难的约束下以及复杂多变的制度和政治环境中进行的。讨论了政策制定者和研究人员之间的沟通过程，包括绘制利益相关者图谱，确定研究沟通计划和定制沟通产品。
结论：尽管研究交流的指导方针和框架是有用的，但不应减损当地团队适应环境的能力。最重要的是当地研究团队的关系和网络。

Translated from English version into Chinese by Pin Yang

CHINESE CENTER FOR DISEASE CONTROL AND PREVENTION
NATIONAL INSTITUTE OF PARASITIC DISEASES

Études sur les maladies à transmission vectorielle : mise en œuvre de stratégies de diffusion des résultats

Thomas Scalway, Mariam Otmani del Barrio et Bernadette Ramirez

Résumé

Introduction: La diffusion des résultats des études portant sur les maladies à transmission vectorielle en Afrique est compliquée à plusieurs égards. Le présent article, qui s’appuie sur les expériences de différents chercheurs recueillies au cours d’un même projet, expose les enseignements pouvant être partagés avec l’ensemble de la communauté scientifique.

Matériels et méthode: Entre 2014 et 2017, cinq équipes interdisciplinaires provenant de sept pays africains ont collaboré dans le cadre d’un projet portant sur les maladies à transmission vectorielle dans le contexte du réchauffement climatique. L’un des principaux objectifs de ce travail était d’influencer l’élaboration des politiques et des programmes à l’aide de résultats de recherche pertinents. Le présent article examine les manières de mettre en pratique les stratégies de diffusion des résultats définies dans les publications scientifiques et les lignes directrices actuelles. Il met en lumière les difficultés rencontrées ainsi que les enseignements qui en ont découlé, et montre que la diffusion de la recherche, qui se déroule dans un environnement institutionnel et politique complexe et changeant, est soumise à d’importantes contraintes. Enfin, les processus de communication entre les décideurs politiques et les chercheurs sont abordés, y compris la cartographie des acteurs, la définition des stratégies de diffusion et la conception des supports de communication.

Résultats: En conclusion, si l’élaboration de lignes directrices et de cadres conceptuels relatifs à la diffusion de la recherche est utile, elle ne saurait se substituer à la capacité d’adaptation des équipes locales. Les relations et les réseaux des équipes de recherche locales s’avèrent ainsi déterminantes.

Translated from English version into French by Théophile Bui, revised by Elise Sahl, through

TRANSLATORS
WITHOUT BORDERS

Исследования по трансмиссивным заболеваниям: осуществление стратегий коммуникации в области научных исследований

Томас Скалвэй (Thomas Scalway), Мариам Отмани дель Баррио (Mariam Otmani del Barrio) и Бернадетт Рамирез (Bernadette Ramirez)

Реферат

Общие сведения: Эффективное распространение результатов исследований по трансмиссивным заболеваниям в Африке является сложной задачей по ряду причин. Учитывая опыт ряда исследователей на протяжении всего срока реализации проекта, в этой статье рассматриваются извлеченные уроки, которыми можно поделиться с более широким научным сообществом.

Основная часть: В период с 2014 по 2017 гг. пять междисциплинарных групп из семи африканских стран сотрудничали в осуществлении проекта, посвященного трансмиссивным заболеваниям в контексте изменения климата. Главная цель этой работы заключалась в оказании влияния на политику и разработку программ с учетом результатов соответствующих исследований. В этой статье рассматриваются возможные способы применения на практике принципов коммуникации в ходе исследований, полученных из литературных источников и действующих руководящих принципов. В докладе освещается ряд проблем и извлеченных уроков, свидетельствующих о том, что коммуникация в области научных исследований осуществляется в сложных

условиях и в неоднозначной, изменчивой институциональной и политической обстановке. Обсуждаются процессы коммуникации между разработчиками политики и исследователями, включая определение круга заинтересованных сторон, определение планов коммуникации в области исследований и адаптацию коммуникационных продуктов.
Выводы: В статье делается вывод о том, что руководящие принципы и базовая структура коммуникации при проведении исследований являются полезными, но они не должны умалять способность местных групп адаптироваться к обстоятельствам. Ключевое значение имеют отношения и сетевая связность местных исследовательских групп.

Translated from English version into Russian by Michael Orlov, revised by Veronika Demeshchyk, through

Investigación de las enfermedades transmitidas por vectores: implementación de estrategias para la comunicación

Thomas Scalway, Mariam Otmani del Barrio y Bernadette Ramirez

Resumen
Antecedentes: La comunicación eficaz de los resultados de la investigación sobre enfermedades transmitidas por vectores en África resulta un desafío por varios motivos. A la vista de las experiencias de diversos investigadores a lo largo de un proyecto, el presente artículo examina las lecciones que pueden ser compartidas con el resto de la comunidad de investigación.
Desarrollo: Entre 2014 y 2017, un conjunto de cinco equipos interdisciplinarios de siete países africanos participó de un proyecto enfocado en las enfermedades trasmitidas por vectores en el contexto del cambio climático. Un objetivo central de este trabajo fue influenciar las políticas y la creación de programas con conclusiones pertinentes de investigaciones. El presente artículo examina cómo los principios de la comunicación de la investigación, derivados de la literatura y de las directrices vigentes, pueden ser aplicados en la práctica. Se destacan varios desafíos y lecciones, demostrando que la comunicación de la investigación se desarrolla entre limitaciones difíciles y en entornos políticos e institucionales complejos e inestables. Se discute el proceso de comunicación entre los encargados de formular políticas y los investigadores, incluidos el mapeo de las partes interesadas, la definición de planes para la comunicación de la investigación y la adaptación de productos de comunicación.
Conclusiones: El artículo concluye que, a pesar de que las directrices y los marcos para la comunicación de la investigación son útiles, esto va en detrimento de la capacidad de los equipos locales para adaptarse a las circunstancias. Las relaciones y redes de los equipos locales de investigación tienen una importancia fundamental.

Translated from English version into Spanish by Pedro Jesús Quintana, revised by Maria Paula Gorgone, through
